# Segmenting Continuous Motions with Hidden Semi-markov Models and Gaussian Processes

**DOI:** 10.3389/fnbot.2017.00067

**Published:** 2017-12-21

**Authors:** Tomoaki Nakamura, Takayuki Nagai, Daichi Mochihashi, Ichiro Kobayashi, Hideki Asoh, Masahide Kaneko

**Affiliations:** ^1^Department of Mechanical Engineering and Intelligent Systems, The University of Electro-Communications, Chofu-shi, Japan; ^2^Department of Mathematical Analysis and Statistical Inference, Institute of Statistical Mathematics, Tachikawa, Japan; ^3^Department of Information Sciences, Faculty of Sciences, Ochanomizu University, Bunkyo-ku, Japan; ^4^Artificial Intelligence Research Center, National Institute of Advanced Industrial Science and Technology, Tsukuba, Japan

**Keywords:** motion segmentation, Gaussian process, hidden semi-Markov model, motion capture data

## Abstract

Humans divide perceived continuous information into segments to facilitate recognition. For example, humans can segment speech waves into recognizable morphemes. Analogously, continuous motions are segmented into recognizable unit actions. People can divide continuous information into segments without using explicit segment points. This capacity for unsupervised segmentation is also useful for robots, because it enables them to flexibly learn languages, gestures, and actions. In this paper, we propose a Gaussian process-hidden semi-Markov model (GP-HSMM) that can divide continuous time series data into segments in an unsupervised manner. Our proposed method consists of a generative model based on the hidden semi-Markov model (HSMM), the emission distributions of which are Gaussian processes (GPs). Continuous time series data is generated by connecting segments generated by the GP. Segmentation can be achieved by using forward filtering-backward sampling to estimate the model's parameters, including the lengths and classes of the segments. In an experiment using the CMU motion capture dataset, we tested GP-HSMM with motion capture data containing simple exercise motions; the results of this experiment showed that the proposed GP-HSMM was comparable with other methods. We also conducted an experiment using karate motion capture data, which is more complex than exercise motion capture data; in this experiment, the segmentation accuracy of GP-HSMM was 0.92, which outperformed other methods.

## 1. Introduction

Human beings typically divide perceived continuous information into segments to enable recognition. For example, humans can segment speech waves into recognizable morphemes. Similarly, continuous motions are segmented into recognizable unit actions. In particular, motions are divided into smaller components called motion primitives, which are used for imitation learning and motion generation (Argall et al., 2009; Lin et al., 2016). It is possible for us to divide continuous information into segments without using explicit segment points. This capacity for unsupervised segmentation is also useful for robots, because it enables them to flexibly learn languages, gestures, and actions.

However, segmentation of time series data is a difficult task. When time series data is segmented, the data points in the sequence must be classified, and each segment's start and end points must be determined. Moreover, each segment affects other segments because of the nature of time series data. Hence, segmentation of time series data requires the exploration of all possible segment lengths and classes. However, this exploration process is difficult; in many studies, the lengths are not estimated explicitly or heuristics are used to reduce computational complexity. Furthermore, in the case of motions, the sequences vary because of dynamic characteristics, even though the same movements are performed. For segmentation of actual human motions, we must address such variations.

**Figure 1:**
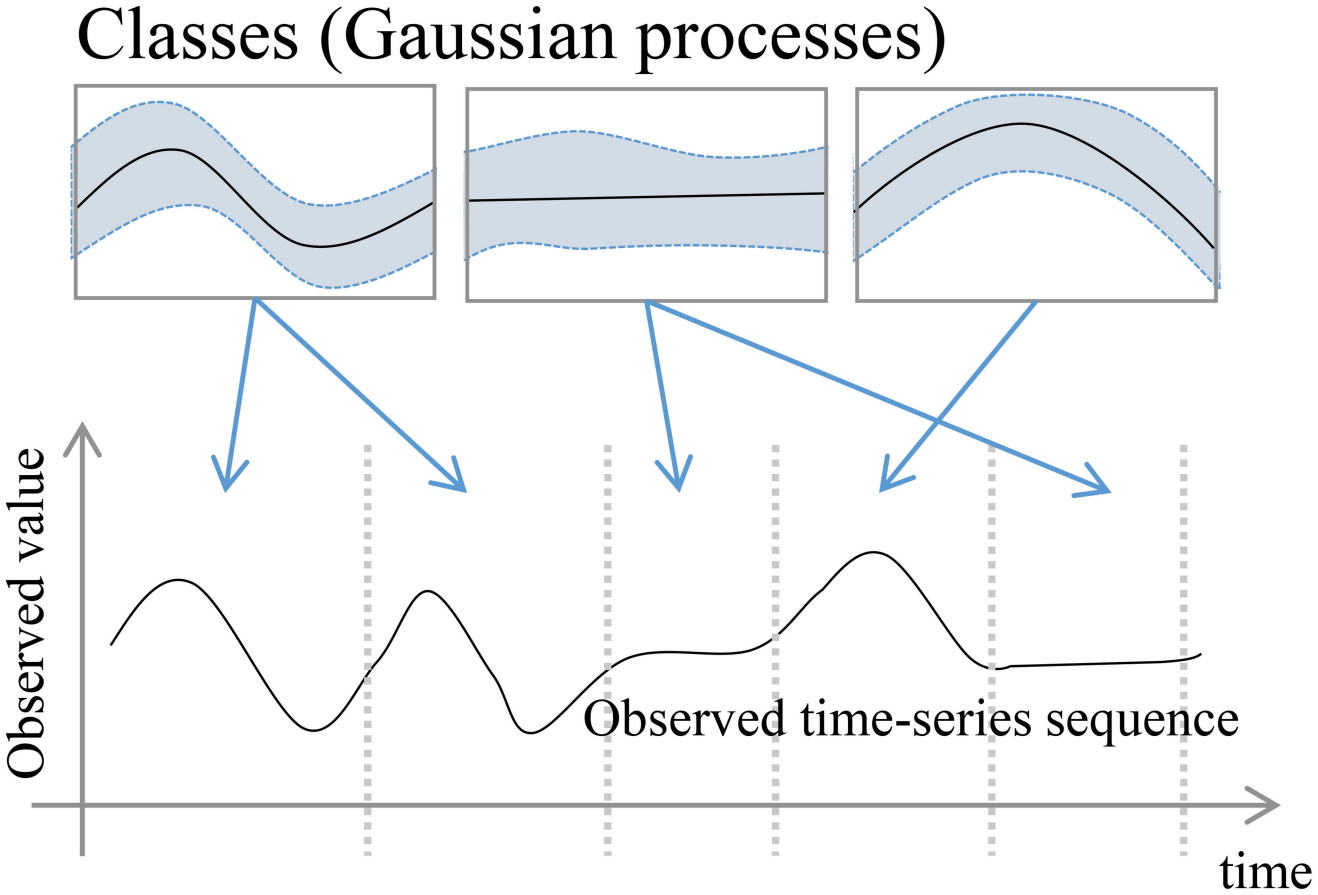
Overview of the proposed GP-HSMM.

In this paper, we propose GP-HSMM (Gaussian process–hidden semi-Markov model), a novel method to divide time series motion data into unit actions by using a stochastic model to estimate their lengths and classes. The proposed method involves a hidden semi-Markov model (HSMM) with a Gaussian process (GP) emission distribution, where each state represents a unit action. Figure 1 shows an overview of the proposed GP-HSMM. The observed time series data is generated by connecting segments generated by each class. The segment points and segment classes are estimated by learning the parameters of the model in an unsupervised manner. Forward filtering-backward sampling (Uchiumi et al., 2015) is used for the learning process; the segment lengths and segment classes are determined by sampling them simultaneously.

## 2. Related work

Various studies have focused on learning motion primitives from manually segmented motions (Gräve and Behnke, 2012; Manschitz et al., 2015). Manschitz et al. proposed a method to generate sequential skills by using motion primitives that are learned in a supervised manner. Gräve et al. proposed segmenting motions using motion primitives that are learned by a supervised hidden Markov model. In these studies, the motions are segmented and labeled in advance. However, we consider that it is difficult to segment and label all possible motion primitives.

Additionally, some studies have proposed unsupervised motion segmentation. However, these studies rely on heuristics. For instance, Wächter et al. have proposed a method to segment human manipulation motions based on contact relations between the end-effectors and objects in a scene (Wachter and Asfour, 2015); in their method, the points at which the end-effectors make contact with an object are determined as boundaries of motions. We believe this method works well in limited scenes; however, there are many motions, such as gestures and dances, in which objects are not manipulated. Lioutikov et al. proposed unsupervised segmentation; however, to reduce computational costs, this technique requires the possible boundary candidates between motion primitives to be specified in advance (Lioutikov et al., 2015). Therefore, the segmentation depends on those candidates, and motions cannot be segmented correctly if the correct candidates are not selected. In contrast, our proposed method does not require such candidates; all possible cutting points are considered by use of forward filtering-backward sampling, which uses the principles of dynamic programming. In some methods (Fod et al., 2002; Shiratori et al., 2004; Lin and Kulić, 2012), motion features (such as the zero velocity of joint angles) are used for motion segmentation. However, these features cannot be applied to all motions. Takano et al. use the error between actual movements and predicted movements as the criteria for specifying boundaries (Takano and Nakamura, 2016). However, the threshold must be manually tuned according to the motions to be segmented. Moreover, they used an HMM that is a stochastic model. We consider such an assumption to be unnatural from the viewpoint of stochastic models, and boundaries should be determined based on a stochastic model. In our proposed method, we do not use such heuristics and assumptions, and instead formulate the segmentation based on a stochastic model.

Fox et al. have proposed unsupervised segmentation for the discovery of a set of latent, shared dynamical behaviors in multiple time series data (Fox et al., 2011). They introduce a beta process, which represents a share of motion primitives in multiple motions, into autoregressive HMM. They formulate the segmentation using a stochastic model, and no heuristics are used in their proposed model. However, in their proposed method, continuous data points that are classified into the same states are extracted as segments, and the lengths of the segments are not estimated. The states can be changed in the short term, and therefore shorter segments are estimated. They reported that some true segments were split into two or more categories, and that those shorter segments were bridged in their experiment. On the other hand, our proposed method classifies data points into states, and uses HSMM to estimate segment lengths. Hence, our proposed method can prevent states from being changed in the short term.

**Figure 2:**
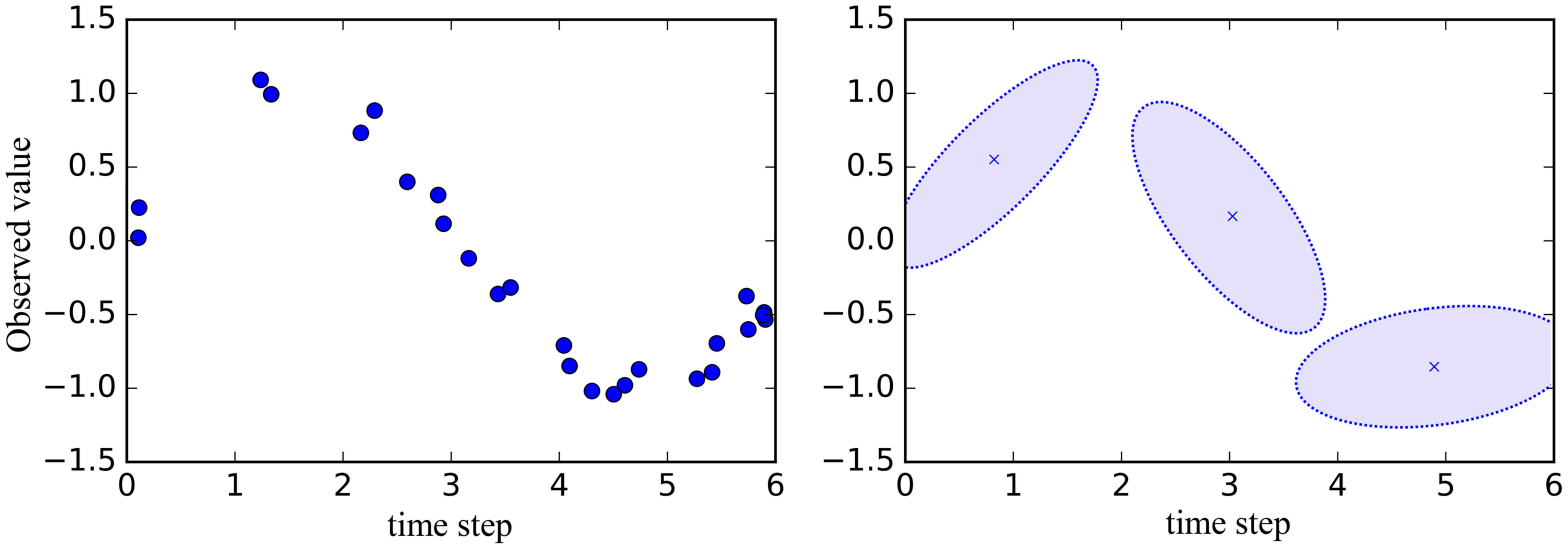
Example of representation of time series data by HMM. **Left:** Data points for learning HMM. **Right:** Mean and standard deviation learned by HMM.

Matsubara et al. proposed an unsupervised segmentation method called AutoPlait (Matsubara et al., 2014). This method uses multiple HMMs, each of which represents a fixed pattern; moreover, transitions between the HMMs are allowed. Therefore, time series data is segmented at points at which the state is changed to another HMM's state. However, we believe that HMMs are too simple to represent complicated sequences such as motions. Figure 2 illustrates an example of representation of time series data by HMM. The graph on the right in Figure 2 represents the mean and standard deviation learned by HMM from data points shown in the graph on the left. HMM represents time series data using only the mean and standard deviation; therefore, details of time series data can be lost. Therefore, we use Gaussian processes, which are non-parametric methods that can represent complex time series data.

The field of natural language processing has also produced literature related to sequence data segmentation. For example, unsupervised morphological analysis has been proposed for segmenting sequence data (Goldwater, 2006; Mochihashi et al., 2009; Uchiumi et al., 2015). Goldwater et al. proposed a method to divide sentences into words by estimating the parameters of a 2-gram language model based on a hierarchical Dirichlet process. The parameters are estimated in an unsupervised manner by Gibbs sampling (Goldwater, 2006). Mochihashi et al. proposed a nested Pitman-Yor language model (NPYLM) (Mochihashi et al., 2009). In this method, parameters of an *n*-gram language model based on the hierarchical Pitman-Yor process are estimated via the forward filtering-backward sampling algorithm. NPYLM can thus divide sentences into words more quickly and accurately than the method proposed in (Goldwater, 2006). Moreover, Uchiumi et al. extended the NPYLM to a Pitman-Yor hidden semi-Markov model (PY-HSMM) (Uchiumi et al., 2015) that can divide sentences into words and estimate the parts of speech (POS) of the words by sampling not only words, but also POS in the sampling phase of the forward filtering-backward sampling algorithm. However, these relevant studies aimed to divide symbolized sequences (such as sentences) into segments, and did not consider analogous divisions in continuous sequence data, such as that obtained by analyzing human motion.

Taniguchi et al. proposed a method to divide continuous sequences into segments by utilizing NPYLM (Taniguchi and Nagasaka, 2011). In their method, continuous sequences are discretized and converted into discrete-valued sequences using the infinite hidden Markov model (Fox et al., 2007). The discrete-valued sequences are then divided into segments by using NPYLM. In this method, motions can be recognized by the learned model, but cannot be generated naively because they are discretized. Moreover, segmentation based on NPYLM does not work well if errors occur in the discretization step.

Therefore, we propose a method to divide a continuous sequence into segments without using discretization. This method divides continuous motions into unit actions. Our proposed method is based on HSMM, the emission distribution of which is GP, which represents continuous unit actions. To learn the model parameters, we use forward filtering-backward sampling, and segment points and classes are sampled simultaneously. However, our proposed method also has limitations. One limitation is that the method requires the number of motion classes to be specified in advance. It is estimated automatically in methods such as (Fox et al., 2011) and (Matsubara et al., 2014). Another limitation is that computational costs are very high, owing to the numerous recursive calculations. We discuss these limitations in the experiments.

## 3. Gaussian process-hidden semi-markov model

**Figure 3:**
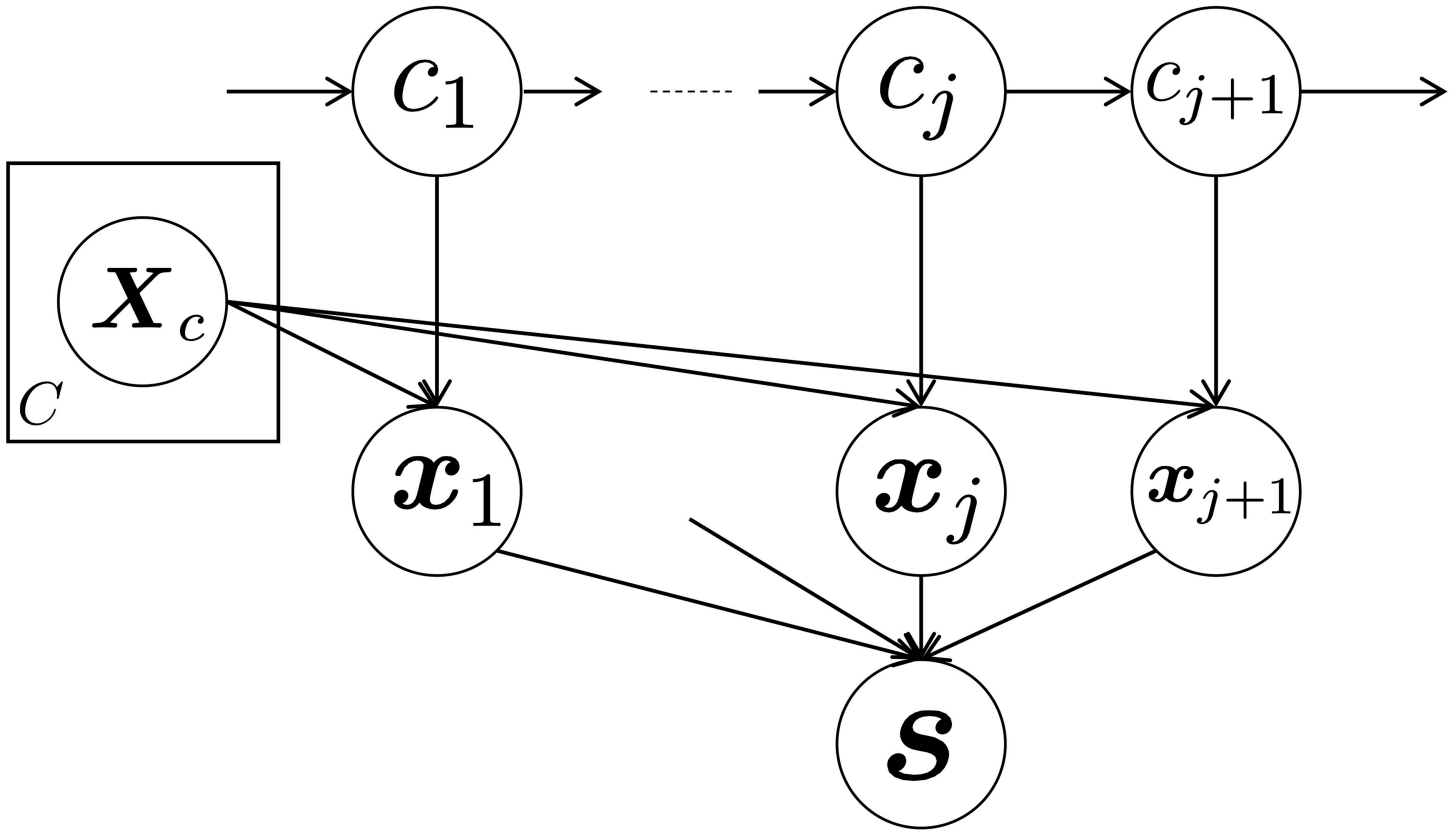
Graphical representation of the proposed GP-HSMM.

Figure 3 shows a graphical representation of the proposed GP-HSMM. In this figure, *c*_*j*_(*j* = 1, 2, · · ·, *J*) denotes classes of segments, and each segment ***x***_*j*_ is generated by a Gaussian process, with parameters denoted by ***X***_*c*_ and given by the following generative process:

(1) $$\begin{array}{rcl} c_{j} & \sim & P\left(c|c_{j-1}\right), \end{array}$$

(2) $$\begin{array}{rcl} x_{j} & \sim & GP\left(x|X_{c_{j}}\right), \end{array}$$

where ***X***_*c*_ represents a set of segments classified into class *c*. Segments are generated by this generative process, and the observed time-series data ***s*** is generated by connecting the segments.

### 3.1. Gaussian process

In this study, we utilize Gaussian process regression, which learns emission *x*_*i*_ of time step *i* in a segment. This makes it possible to represent each unit action as part of a continuous trajectory. If we obtain pairs (***i***, ***X***_*c*_) of emissions *x*_*i*_ of time step *i* of segments belonging to the same class *c*, a predictive distribution whereby the emission of time step *i* becomes *x* follows a Gaussian distribution.

(3) $$\begin{array}{r} p\left(x|i,X_{c},i\right)\propto N\left(k^{T}C^{-1}i,c-k^{T}C^{-1}k\right), \end{array}$$

where *k*(·, ·) represents the kernel function and ***C*** is a matrix whose elements are

(4) $$\begin{array}{r} C\left(i_{p},i_{q}\right)=k\left(i_{p},i_{q}\right)+\beta^{-1}\delta_{pq}. \end{array}$$

**Figure 4:**
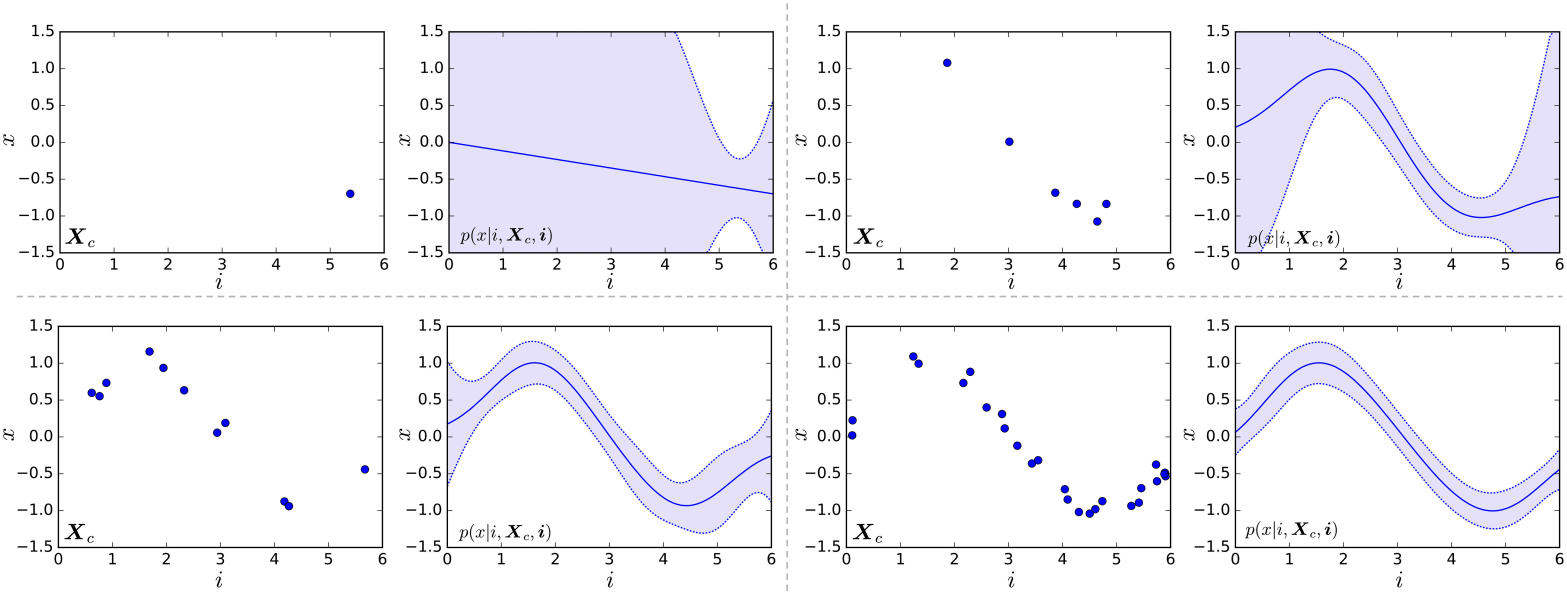
Examples of Gaussian processes. Left graph in each pair of graphs represents learning data points (***i***, ***X***_*c*_). Right graph shows the learned probabilistic distribution *p*(*x*|*i*, ***X***_*c*_, ***i***); the solid line represents the mean, and the blue region represents the range of standard deviation.

β is a hyperparameter that represents noise in the observation. In Equation (3), ***k*** is a vector containing the elements *k*(*i*_*p*_, *i*), and *c* is a scalar value *k*(*i, i*). Using the kernel function, GP can learn a time-series sequence that contains complex changes. We use the following Gaussian kernel, which is generally used for Gaussian process regression:

(5) $$\begin{array}{r} k\left(i_{p},i_{q}\right)=\theta_{0}\exp\left(-\frac{1}{2}||i_{p}-i_{q}|{|}^{2}+\theta_{2}+\theta_{3}i_{p}i_{q}\right), \end{array}$$

where θ_*_ represents parameters of the kernel. Figure 4 shows examples of Gaussian processes. The left graph in each pair of graphs represents learning data points (***i***, ***X***_*c*_), and the right graph shows the learned probabilistic distribution *p*(*x*|*i*, ***X***_*c*_, ***i***). One can see that the standard deviation decreases with an increase in the number of learning data points. If the emission of time step *i* is multidimensional vector ***x*** = (*x*_0_, *x*_1_, · · ·), we assume that each dimension is generated independently, and a predictive distribution $GP\left(x|X_{c}\right)$ is computed as follows:

(6) $$\begin{array}{rcl} GP\left(x|X_{c}\right) & = & p\left(x_{0}|i,X_{c,0},i_{c}\right)\\ \;\times p\left(x_{1}|i,X_{c,1},i_{c}\right)\\ \;\times p\left(x_{2}|i,X_{c,2},i_{c}\right)\cdot \cdot \cdot . \end{array}$$

Based on this probability, similar segments can be classified into the same class.

### 3.2. Learning of GP-HSMM

#### 3.2.1. Blocked gibbs sampler

Segments and classes of segments in the observed sequences are estimated based on dynamic programming and sampling. For efficient sampling, we use the blocked Gibbs sampler, which samples segments and their classes in an observed sequence. In the initialization phase, all observed sequences are first randomly divided into segments. Segments ***x***_*nj*_(*j* = 1, 2, · · ·, *J*_*n*_) in observed sequence ***s***_*n*_ are then removed from the learning data, and parameter ***X***_*c*_ of the Gaussian process and transition probability *P*(*c*|*c*′) of HSMM are updated. Segments ***x***_*nj*_(*j* = 1, 2, · · ·, *J*_*n*_) and their classes *c*_*nj*_(*j* = 1, 2, · · ·, *J*_*n*_) are then estimated as follows:

(7) $$\begin{array}{r} \left(x_{n1},\cdot \cdot \cdot ,x_{nJ_{n}}\right),\left(c_{n1},\cdot \cdot \cdot ,c_{nJ_{n}}\right)\sim P\left(X,\text{c}|s_{n}\right), \end{array}$$

where ***X*** is a set of segments into which ***s***_*n*_ is divided, and ***c*** denotes classes of the segments. To carry out this sampling efficiently, the probability of all possible segments ***X*** and classes ***c*** must be computed; however, these probabilities are difficult to compute simply because the number of potential combinations is very large. Thus, we utilize forward filtering-backward sampling, which we presently explain. After sampling ***x***_*nj*_ and *c*_*nj*_, parameter ***X***_*c*_ of the Gaussian process and transition probability *P*(*c*|*c*′) of HSMM are updated by adding them to the learning data. The segments and parameters of Gaussian processes are optimized alternately by iteratively performing the above procedure. Algorithm 1 shows the pseudocode of the blocked Gibbs sampler. *N*_*c*_*nj*__ and *N*_*c*_*nj*_, *c*_*n, j*+1__ represent parameters for computing the transition probability in Equation (10).

**Table:** **Algorithm 1** Blocked Gibbs Sampler

1: // Iterate the following procedure until convergence
2: **for** *n* = 1 to *N* **do**
3: **for** *j* = 1 to *J*_*n*_ **do**
4: *N*_*c*_*nj*__− = 1
5: *N*_*c*_*nj*_, *c*_*n, j*+1__− = 1
6: **if** *j* ≠ 0 **then**
7: Delete segments ***x***_*nj*_ from ***X***_*c*_*nj*__
8: **end if**
9: **end for**
10:
11: // Sample segments and their classes
12: (***x***_*n*1_, · · ·, ***x***_*nJ*_*n*__), (*c*_*n*1_, · · ·, *c*_*nJ*_*n*__) ~ *P*(***X***, ***c***|***s***_*n*_)
13:
14: **for** *j* = 1 to *J*_*n*_ **do**
15: *N*_*c*_*nj*__++
16: *N*_*c*_*nj*_, *c*_*n, j*+1__++
17: **if** *j* ≠ **then**
18: Add segments ***x***_*nj*_ into ***X***_*c*_*nj*__
19: **end if**
20: **end for**
21: **end for**

#### 3.2.2. Forward filtering-backward sampling

**Figure 5:**
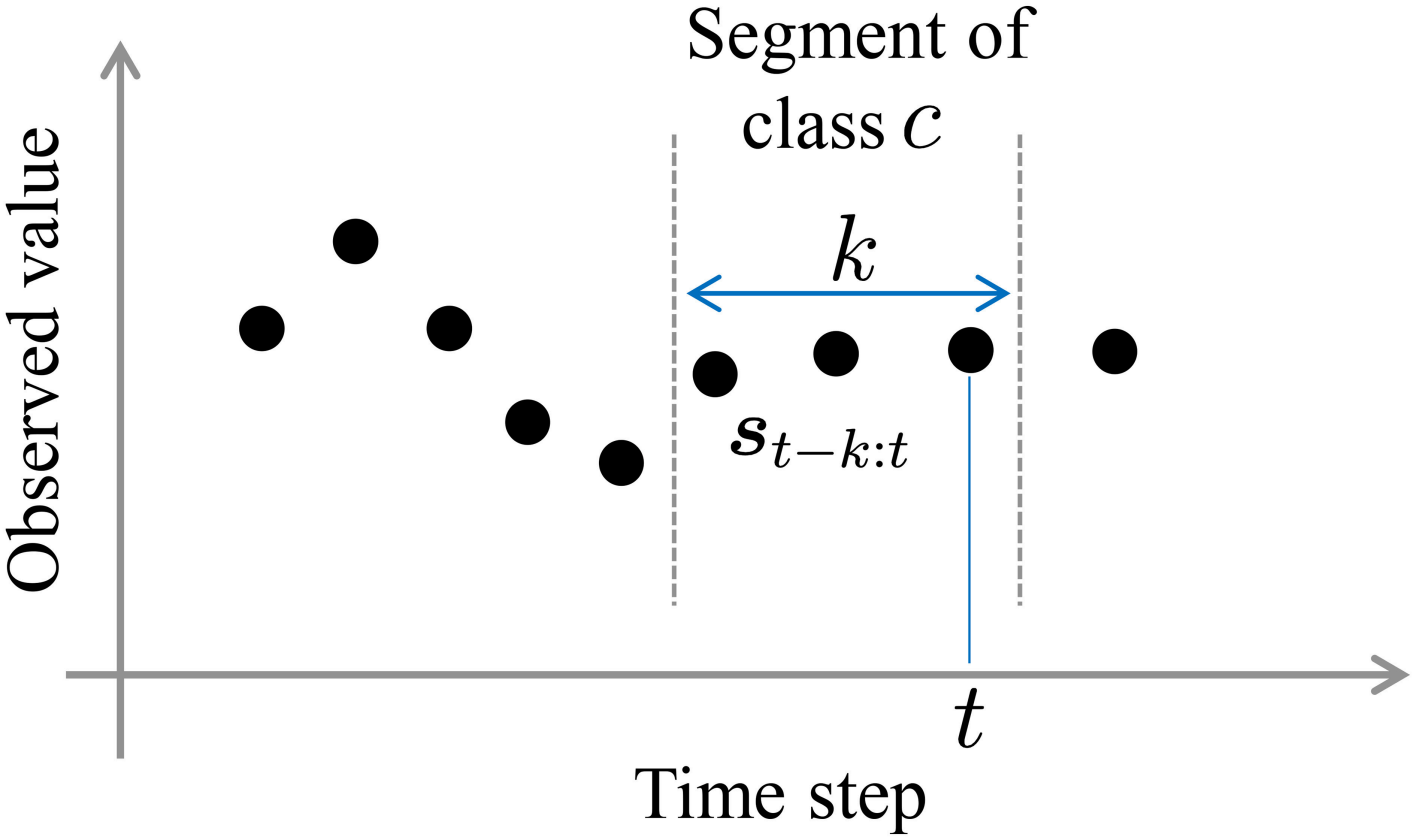
A segment whose probability is computed during forward filtering.

**Figure 6:**
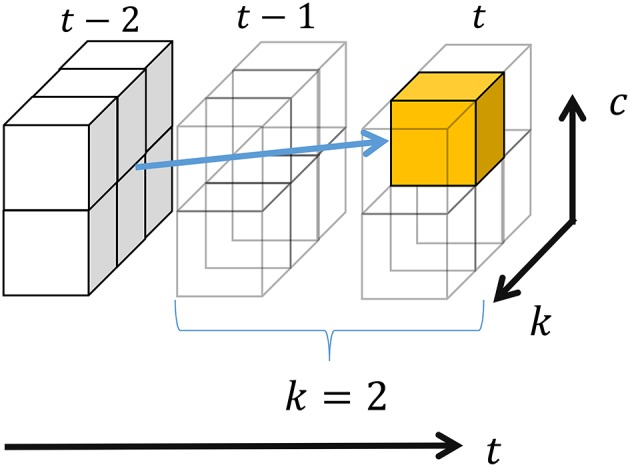
Recursive computation in forward filtering.

In this study, we regard segments and their classes as latent variables that are sampled by forward filtering-backward sampling (Algorithm 2). In forward filtering, as shown in Figure 5, the probability that *k* samples ***s***_*t*−*k* : *t*_ prior to time step *t* in observed sequence ***s*** form a segment, and that the resulting segment belongs to class *c*, is computed as follows:

(8) $$\begin{array}{rcl} \alpha \left[t\right]\left[k\right]\left[c\right] & = & P\left(s_{t-k:t}|X_{c}\right)\\ \;\times \overset{K}{\underset{k^{\prime }=1}{\sum }}\overset{C}{\underset{c^{\prime }=1}{\sum }}p\left(c|c^{\prime }\right)\alpha \left[t-k\right]\left[k^{\prime }\right]\left[c^{\prime }\right], \end{array}$$

where *C* and *K* denote the number of classes and the maximum length of segments, respectively. *P*(***s***_*t*−*k* : *t*_|***X***_*c*_) represents the probability that ***s***_*t*−*k* : *t*_ is generated from a class *c*; this is computed as follows:

(9) $$\begin{array}{r} P\left(s_{t-k:t}|X_{c}\right)=GP\left(s_{t-k:t}|X_{c}\right)P_{len}\left(k|\lambda \right). \end{array}$$

where *P*_*len*_(*k*|λ) represents a Poisson distribution with a mean parameter λ; this corresponds to the distribution of the segment lengths. *p*(*c*|*c*′) in Equation (8) represents a transition probability computed as follows:

(10) $$\begin{array}{r} p\left(c|c^{\prime }\right)=\frac{N_{c^{\prime }c}+\alpha }{N_{c^{\prime }}+C\alpha }, \end{array}$$

where $N_{c^{\prime }}$ and $N_{c^{\prime }c}$ denote the number of segments whose classes are *c*′ and the number of transitions from *c*′ to *c*, respectively, and *k*′ and *c*′ respectively denote the length and class of the segment preceding ***s***_*t*−*k* : *t*_; these are marginalized out in Equation (8). Moreover, α[*t*][*k*][*] = 0 if *t* − *k* < 0, and α[0][0][*] = 1.0. All elements of α[*][*][*] in Equation (8) can be recursively computed from α[1][1][*] by dynamic programming. Figure 6 depicts the computation of a three-dimensional array α[*t*][*k*][*c*]. In this example, the probability that two samples before time step *t* become a segment is computed; the resulting segment would be assigned to class two. Hence, samples at *t* − 1 and *t* become a segment, and all the segments whose end point is *t* − 2 can potentially transit to this segment. α[*t*][2][2] can be computed by marginalizing out these possibilities.

**Table:** **Algorithm 2** Forward filtering-backward sampling

1: // Forward filtering
2: **for** *t* = 1 to *T* **do**
3: **for** *k* = 1 to *K* **do**
4: **for** *c* = 1 to *C* **do**
5: Compute α[*t*][*k*][*c*]
6: **end for**
7: **end for**
8: **end for**
9:
10: // Backward sampling
11: *t* = *T, j* = 1
12: **while** *t* > 0 **do**
13: *k, c* ~ α[*t*][*k*][*c*]
14: ***x***_*j*_ = ***s***_*t*−*k* : *t*_
15: *c*_*j*_ = *c*
16: *t* = *t* − *k*
17: *j* = *j* + 1
18: **end while**
19: return (***x***_*J*_*n*__, ***x***_*J*_*n*_−1_, · · ·, ***x***_1_), (*c*_*J*_*n*__, *c*_*J*_*n*_−1_, · · ·, *c*_1_)

Finally, segment ***x***_*j*_ and its class are determined by backward sampling length *k* and class *c* of the segment, based on forward probabilities in α. From *t* = *T*, length *k*_1_ and class *c*_1_ are determined according to *k*_1_, *c*_1_ ~ α[*T*][*k*][*c*], and ***s***_*T*−*k*_1_ : *T*_ becomes a segment whose class is *c*_1_. Then, length *k*_2_ and class *c*_2_ of the next segment are determined according to *k*_2_, *c*_2_ ~ α[*T* − *k*_1_][*k*][*c*]. By iterating this procedure until *t* = 0, the observed sequence can be divided into segments and their classes can be determined.

## 4. Experiments

We conducted experiments to confirm the validity of the proposed method. We used two types of motion capture data: (1) data from the CMU motion capture dataset (CMU, 2009), and (2) data containing karate motions.

### 4.1. Segmentation of exercise motions

**Figure 7:**
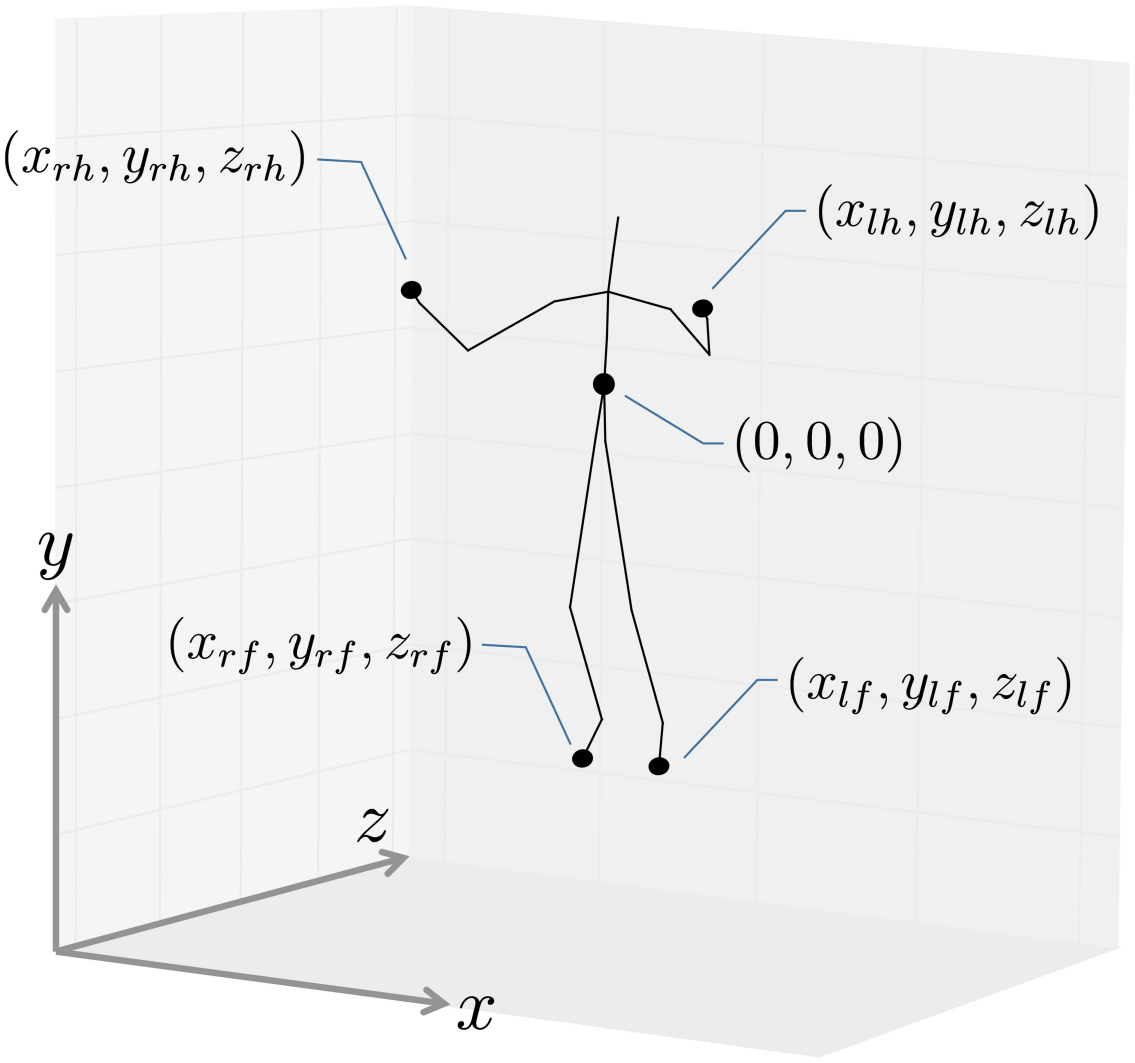
Coordinate system used in the experiments.

We first applied our proposed method to CMU motion capture data containing several exercise routines. The CMU motion capture data was captured using a Vicon motion capture system, and positions and angles of 31 body parts are available. The dataset contains 2605 trials in six categories and 23 subcategories, and motions in each subcategory were performed by one or a few subjects. In this experiment, three sequences from subject 14 in the general exercise and stretching category were used, and include running, jumping, squats, knee raises, reach out stretches, side stretches, body twists, up and down movements, and toe touches. To reduce computational cost, we downsampled from 120 frames per second to 4 frames per second. Figure 7 shows the coordinate system of motion capture data used in this experiment; two-dimensional frontal views of the left hand (*x*_*lh*_, *y*_*lh*_), right hand (*x*_*rh*_, *y*_*rh*_), left foot(*x*_*lf*_, *y*_*lf*_), and right foot (*x*_*rf*_, *y*_*rf*_) were used. Therefore, each frame was represented by eight dimensional vectors: (*x*_*lh*_, *y*_*lh*_, *x*_*rh*_, *y*_*rh*_, *x*_*lf*_, *y*_*lf*_, *x*_*rf*_, *y*_*rf*_). Because GP-HSMM requires the number of classes to be specified in advance, we set it to eight.

**Figure 8:**
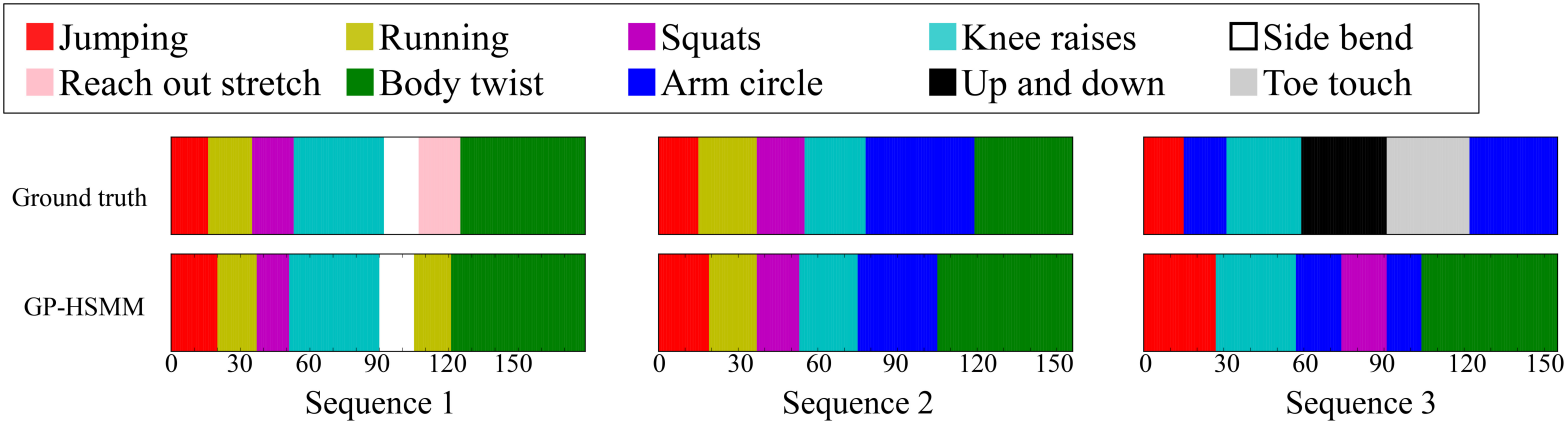
Segmentation results of CMU motion capture data.

**Figure 9:**
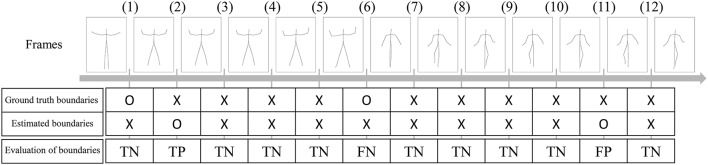
Example of segmentation evaluation. Estimated boundaries are evaluated as true positive (TP), true negative (TN), false positive (FP), or false negative (FN).

Figure 8 shows the results of the segmentation. The horizontal axis represents the frame number, and the colors represent motion classes into which each segment was classified. The segments were classified into seven classes out of eight. Table 1 shows the accuracy of the segmentation. We computed the following normalized Hamming distance between the unsupervised segmentation and the ground truth:

(11) $$\begin{array}{r} ND\left(c,\bar{c}\right)=\frac{D\left(c,\bar{c}\right)}{\left|\bar{c}\right|}, \end{array}$$

where ***c*** and $\bar{c}$ represent sequences of estimated motion classes and true motion classes, $D\left(c,\bar{c}\right)$ is the Hamming distance between two sequences, and $\left|\bar{c}\right|$ represents the length of the sequence. Therefore, the normalized Hamming distance ranges from 0 to 1; lower Hamming distances indicate more accurate segmentation. In this experiment, the Hamming distance was 0.33, which is comparable with the BP-HMM reported in (Fox et al., 2011). However, they also reported that some segments were split into two or more categories, and that those shorter segments were bridged. In contrast, we performed no such modifications, and Figure 8 shows that there are no shorter segments. We also computed the precision, recall, and F-measure of the segmentation. To compute them, estimated boundaries of segments are evaluated as true positive (TP), true negative (TN), false positive (FP), or false negative (FN). Figure 9 shows an example of segmentation evaluation. We considered the estimated boundary to be TP if it was within true boundary ± four frames, as shown in Figure 9(2). If the ground truth boundary has no corresponding estimated boundary as shown in Figure 9(6), it was considered as FN. Conversely, if the estimated boundary has no corresponding ground truth boundary as shown in Figure 9(11), it was considered as FP. From these evaluations, the precision, recall, and F-measure of the segmentation are computed as follows:

(12) $$\begin{array}{rc} P & =\frac{N_{TP}}{N_{TP}+N_{FP}}, \end{array}$$

(13) $$\begin{array}{rc} R & =\frac{N_{TP}}{N_{TP}+N_{FN}}, \end{array}$$

(14) $$\begin{array}{rc} F & =\frac{2PR}{P+R}, \end{array}$$

where *N*_*TP*_, *N*_*FP*_, and *N*_*TN*_ represent the number of points assessed as TP, FP, and FN. The F-measure of the segmentation was 0.81, and this fact indicates that GP-HSMM can estimate boundaries reasonably. This is because GP-HSMM estimates the length of segments as well as the classes of segments.

**Table 1:** Segmentation accuracy of CMU motion capture data.

**Hamming distance**	**Precision**	**Recall**	**F-measure**
0.33	0.81	0.81	0.81

Moreover, Figure 8 shows that most false segmentations are in sequence 3. This is because “up and down” and “toe touch” motions are included only in sequence 3, and GP-HSMM was not able to extract patterns that occur infrequently. However, this problem is not limited to GP-HSMM, and it is generally difficult for any learning method to extract infrequent patterns. The Hamming distance, which was computed only from sequence 1 and sequence 2, was 0.15. This result shows that GP-HSMM can accurately estimate segments that appear multiple times in a sequence.

### 4.2. Segmentation of karate motion

**Figure 10:**
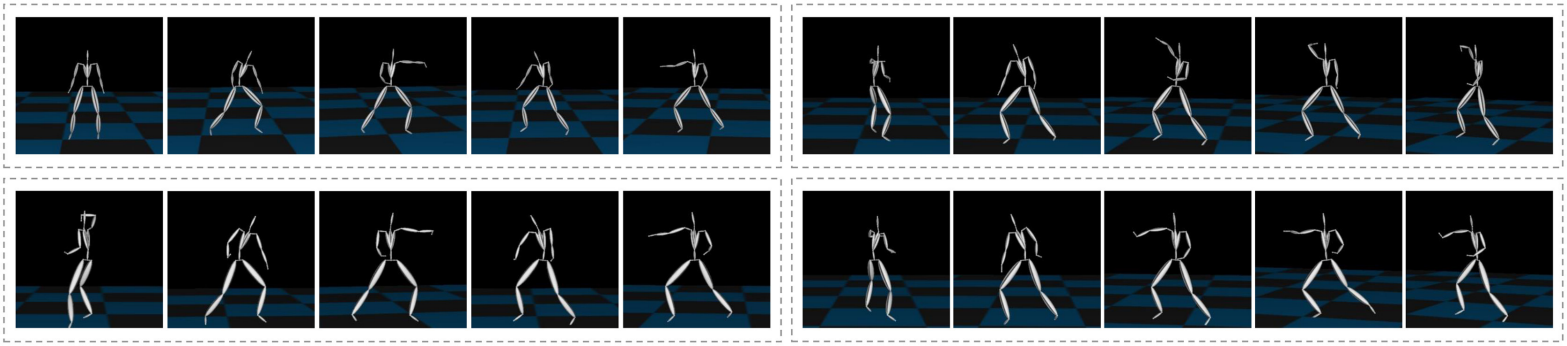
Motion capture data of karate motions.

**Figure 11:**
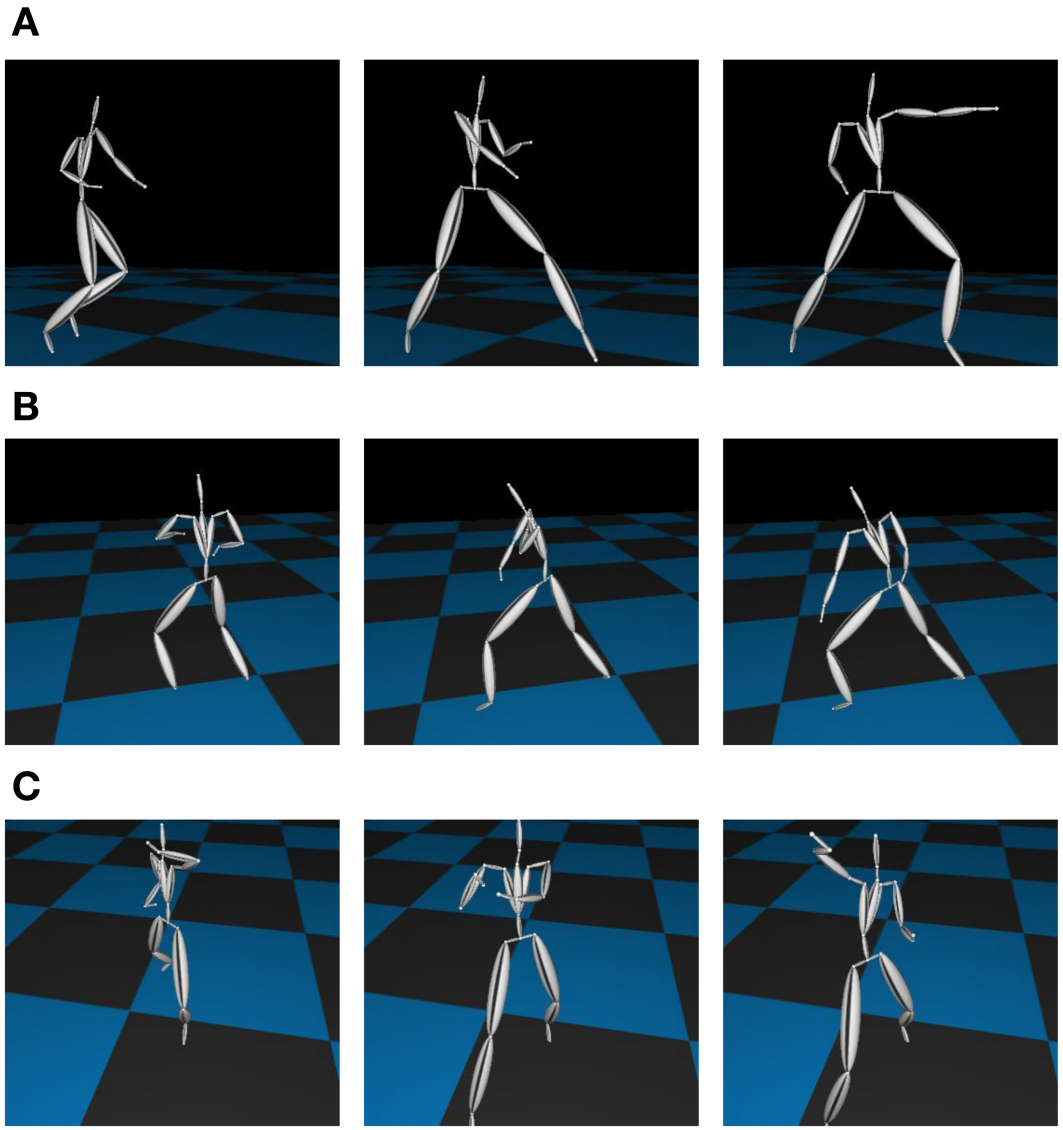
Basic motions in Kata: **(A)** Left punch. **(B)** Left lower guard. **(C)** Right upper guard.

We then applied our proposed method to more complex motion capture data, which consisted of the basic motions of karate (called kata in Japanese)^1^ as shown in Figure 10 from the motion capture library Mocapdata.com^2^. There are fixed motion patterns (punches or guards) in kata, and it is easy to form a ground truth for the segmentation. However, there might be shorter motion patterns, and GP-HSMM might be able to find those motion patterns if the number of classes is set to a larger number. Moreover, it is possible for GP-HSMM to discover patterns that cannot be labeled by humans, and GP-HSMM has the potential to analyze unlabeled time series data. However, in this experiment, we must evaluate the proposed method quantitatively, and fixed motion patterns (punches or guards) labeled by a human expert are used as ground truth. The type of kata we used was called heian 1, which is the most basic form of kata consisting of punches, lower guard, and upper guard (Tsuki, Gedanbarai, and Joudanuke in Japanese). Figure 11 shows the basic movements used in heian 1. We divided this motion sequence into four parts, for use as four motion sequences to apply the blocked Gibbs sampler. Each motion sequence consisted of the following actions:

1. Left lower guard, right punch, right lower guard, and left punch.
2. Left lower guard, right upper guard, left upper guard, and right upper guard.
3. Left lower guard, right punch, right lower guard, and left punch.
4. Left lower guard, right punch, left punch, and right punch

By way of its preprocessing, as shown in Figure 7, the motion capture data was converted into motions with the body facing forward with a center of (0,0,0). To reduce computational cost, we downsampled the motion capture data from 30 frames per second to 15 frames per second, and used two-dimensional left-hand positions (*x*_*lh*_, *y*_*lh*_) and right-hand positions (*x*_*rh*_, *y*_*rh*_) in the frontal view, as shown in Figure 7. To compare our method with others, we used segmentation based on HDP-HMM (Beal et al., 2001) and segmentation based on NPYLM and HDP-HMM (Taniguchi and Nagasaka, 2011), where NPYLM (Mochihashi et al., 2009) divides sequences discretized by HDP-HMM. In addition, we compared our method with BP-HMM (Fox et al., 2011) and AutoPlait (Matsubara et al., 2014).

**Figure 12:**
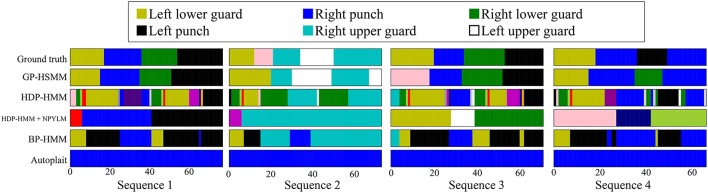
Results of segmentation and classification for each method.

**Figure 13:**
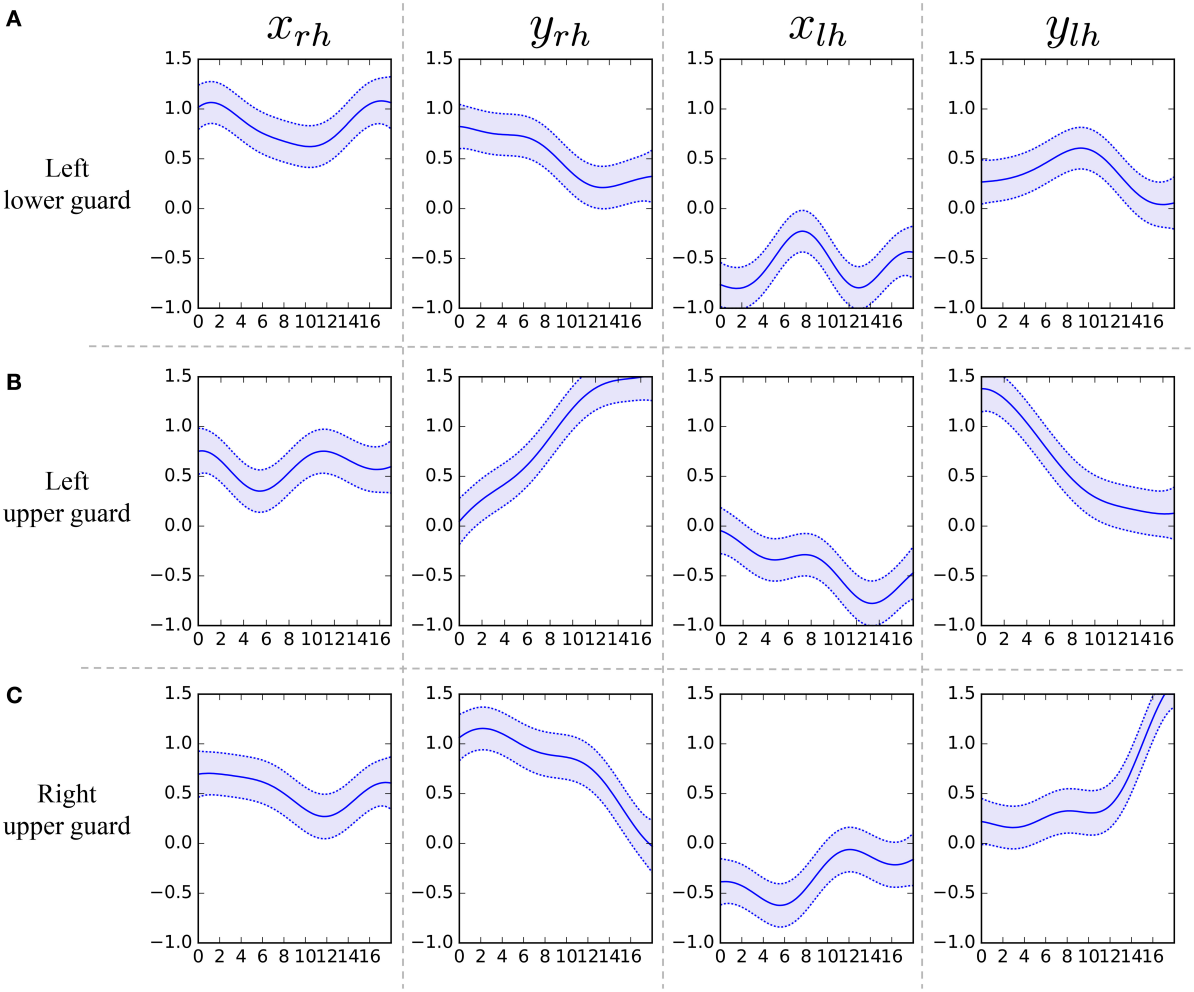
Learned Gaussian processes for left lower guard, left upper guard, and right upper guard.

Figure 12 shows the segmentation results. The horizontal axis represents the frame number, and the colors represent motion classes into which each segment was classified. The figure shows that HDP-HMM estimated shorter segments than the ground truth. This occurred because the emission distribution of HDP-HMM is a Gaussian distribution, which cannot represent continuous trajectories. Moreover, the result produced by segmentation, in which NPYLM divided sequences discretized by HDP-HMM, yielded longer segments. Moreover, NPYLM cannot extract fixed patterns of sequences. This is because the sequences discretized by HDP-HMM included noise and, therefore, NPYLM was unable to find a pattern in them. It was also difficult for BP-HMM to estimate correct segments, and some shorter segments were present. Further, AutoPlait could not find any segments in the karate motion sequences. We believe this occurred because HMMs are too simple to model complex motions. On the contrary, we use Gaussian processes that make it possible to model complex sequences. Table 2 shows the segmentation accuracy of each method. We considered the estimated boundary to be correct if it was within true boundary ± five frames. The F-measure of the proposed method was 0.92, which indicates that GP-HSMM can estimate boundaries accurately. The results show that GP-HSMM outperforms the other methods. Figure 13 shows the learned Gaussian process. *y*_*rh*_ in Figure 13A, which represents the height of the left hand, is decreased, which indicates the motion where the left hand is dropped for the lower guard. In contrast, *y*_*rh*_ in Figure 13B is increased, which indicates the motion where the left hand is raised for the upper guard. Conversely, *y*_*lh*_ in Figure 13C is increased for the right upper guard. From this result, we can see that characteristics of motions can be learned by Gaussian processes.

**Table 2:** Segmentation accuracy of karate motions.

	**Hamming distance**	**Precision**	**Recall**	**F-measure**
GP-HSMM	0.21	0.92	0.92	0.92
HDP-HMM	0.47	0.12	0.54	0.19
HDP-HMM + NPYLM	0.61	0.00	0.00	0.00
BP-HMM	0.49	0.13	0.23	0.16
AutoPlait	0.76	0.00	0.00	0.00

Moreover, the motions were classified into seven classes, although we set the number of classes to eight. This result indicates that the number of classes can be estimated to a certain extent, if a number closer to the correct number is given. However, a smaller number leads to under-segmentation and misclassification, and a much larger number leads to over-segmentation. This is a limitation of the current GP-HSMM, and we believe it can be solved by introducing a non-parametric Bayesian model.

Computational cost is another limitation of GP-HSMM. Table 3 shows the computational time required to segment karate motion. HMM-based methods such as HDP-HMM, BP-HMM, and AutoPlait are relatively faster. In particular, AutoPlait is the fastest because it uses a single scan algorithm proposed in (Matsubara et al., 2014) to find boundaries, and it has been demonstrated that AutoPlait can detect meaningful patterns from large datasets. In contrast, our proposed GP-HSMM is much slower than other methods, and cannot process such large datasets. This is another limitation of the proposed method.

**Table 3:** Computational time of each method.

	**Time (s)**
GP-HSMM	248
HDP-HMM	1.99
HDP-HMM + NPYLM	18.2
BP-HMM	3.37
AutoPlait	0.31

## 5. Conclusion

In this paper, we proposed a method for motion segmentation based on a hidden semi-Markov model (HSMM) with a Gaussian process (GP) emission distribution. By employing HSMM, segment classes and their lengths can be estimated. Moreover, a forward filtering-backward sampling algorithm is used to estimate the parameters of GP-HSMM; this makes it possible to efficiently search for all possible segment lengths and classes. The experimental results showed that the proposed method can accurately segment motion capture data. Although motions that occurred in the sequences a single time were difficult to segment correctly, motions that occurred a few times could be segmented with higher accuracy.

However, some issues remain in the current GP-HSMM. The most significant problem is that GP-HSMM requires the number of classes to be specified in advance. We believe this value can be estimated by utilizing a non-parametric Bayesian model. We are planning to introduce a stick-breaking process as a prior distribution of the transition matrix, and beam sampling for parameter estimation; these techniques are utilized in Beal et al. (2001). Another problem is computational cost. The computational cost to learn a Gaussian process is *O*(*n*^3^), where *n* denotes the number of data points classified in the GP. To overcome this problem, efficient computation methods have been proposed (Nguyen-Tuong et al., 2009; Okadome et al., 2014), and we will consider introducing these methods into GP-HSMM.

## Author contributions

ToN, TaN, DM, IK, and HA conceived of the presented idea. ToN, TaN, and DM developed the theory and performed the computations. IK and HA verified the theory and the analytical methods. ToN wrote the manuscript with support from TaN and MK. IK and HA supervised the project. All authors discussed the results and contributed to the final manuscript.

### Conflict of interest statement

The authors declare that the research was conducted in the absence of any commercial or financial relationships that could be construed as a potential conflict of interest.

## Footnotes

^1^https://mocapdata.blob.core.windows.net/freemotions/karate.zip

^2^http://www.mocapdata.com/
